# Supplementary material for: A Likelihood Approach for Real-Time Calibration of Stochastic Compartmental Epidemic Models
Source: PLoS Comput Biol. 2017 Jan 17;13(1):e1005257. doi: 10.1371/journal.pcbi.1005257 (PMC5240920; doi:10.1371/journal.pcbi.1005257)
Supplement: S1 File — (TAR.GZ) [file pcbi.1005257.s014.tar.gz › HSPH_Online-SI-Revision/Additions/EnKF08/n100-extreme-SEITR/n100-extreme-SEITR_EnKF2_table0.pdf]

1	newly 3 weeks spec	6372.	1734.13	{324., 2245.}	87.0284	0.869554	newly 3 weeks cumul	27578.	6263.75	{2524., 11490.}	79.6441	0.780881	newly 5 weeks spec	2747.	1067.48	{87., 1841.}	91.3156	0.932373	newly 5 weeks cumul	34544.	8744.6	{2886., 16138.}	80.7411	0.781486	newly 51 weeks cumul	38811.	12597.8	{3069., 25701.}	79.4052	0.744249
2	newly 3 weeks spec	7321.	4005.41	{863., 17914.}	81.5581	0.600743	newly 3 weeks cumul	36564.	24085.5	{6907., 109168.}	81.165	0.492398	newly 5 weeks spec	2664.	1122.74	{46., 2515.}	65.1652	0.73006	newly 5 weeks cumul	43633.	27257.9	{8296., 119158.}	78.7557	0.490736	newly 58 weeks cumul	47260.	29175.7	{8987., 120697.}	76.1354	0.478892
3	newly 3 weeks spec	8747.	375.58	{28., 518.}	96.3116	1.91937	newly 3 weeks cumul	40258.	2393.58	{209., 3364.}	94.0544	1.48995	newly 5 weeks spec	3413.	150.29	{1., 134.}	98.3179	2.50441	newly 5 weeks cumul	49075.	2781.63	{236., 3754.}	94.3938	1.5498	newly 60 weeks cumul	54208.	3021.09	{240., 3922.}	94.6665	1.58572
4	newly 3 weeks spec	8496.	1397.5	{70., 2240.}	86.9633	1.12187	newly 3 weeks cumul	36998.	9762.38	{5093., 10017.}	80.0912	0.700214	newly 5 weeks spec	3304.	621.78	{0., 1096.}	91.3626	1.66572	newly 5 weeks cumul	45863.	11266.6	{5893., 13511.}	80.7774	0.731748	newly 58 weeks cumul	50745.	13233.4	{6043., 20877.}	80.3771	0.735177
5	newly 3 weeks spec	5120.	1090.18	{30., 370.}	99.7324	1.60365	newly 3 weeks cumul	31803.	6199.58	{332., 6187.}	93.6432	1.28357	newly 5 weeks spec	1318.	307.72	{1., 86.}	105.948	2.09932	newly 5 weeks cumul	35687.	7090.88	{379., 6318.}	94.0328	1.30948	newly 60 weeks cumul	37045.	7460.1	{391., 6331.}	94.0106	1.31814
6	newly 3 weeks spec	4778.	964.41	{6., 454.}	103.612	1.94733	newly 3 weeks cumul	31868.	3896.63	{77., 6262.}	93.1416	1.59269	newly 5 weeks spec	1057.	632.92	{0., 159.}	141.067	2.2636	newly 5 weeks cumul	35197.	5370.2	{82., 6262.}	95.8891	1.6059	newly 59 weeks cumul	35989.	6438.78	{83., 6262.}	97.7193	1.60305
7	newly 3 weeks spec	7357.	1245.21	{280., 1958.}	88.8801	1.06904	newly 3 weeks cumul	28195.	5315.05	{1304., 6525.}	86.5294	0.987319	newly 5 weeks spec	3977.	580.	{42., 1020.}	87.6047	1.16344	newly 5 weeks cumul	37670.	6699.05	{1807., 9954.}	85.5144	0.992163	newly 51 weeks cumul	47209.	9008.3	{2119., 12703.}	83.999	0.96652
8	newly 3 weeks spec	7216.	1403.98	{13., 2794.}	81.0535	1.12737	newly 3 weeks cumul	32722.	11396.	{3381., 15161.}	80.3568	0.694471	newly 5 weeks spec	3068.	434.46	{0., 1198.}	85.839	1.64832	newly 5 weeks cumul	40537.	12594.2	{3470., 18466.}	79.7456	0.727841	newly 58 weeks cumul	46063.	13256.9	{3576., 21056.}	79.1608	0.749997
9	newly 3 weeks spec	5164.	753.7	{61., 414.}	96.7498	1.47222	newly 3 weeks cumul	32212.	5347.55	{1085., 5505.}	90.7379	1.15016	newly 5 weeks spec	1330.	225.48	{2., 59.}	101.853	1.91621	newly 5 weeks cumul	36196.	5982.81	{1155., 5589.}	90.7871	1.1759	newly 60 weeks cumul	37584.	6297.6	{1168., 5597.}	90.3886	1.18378
10	newly 3 weeks spec	8694.	3138.86	{129., 14448.}	87.1693	1.15155	newly 3 weeks cumul	33638.	20054.7	{1555., 83401.}	102.299	0.909877	newly 5 weeks spec	4707.	1059.57	{4., 4803.}	89.1304	1.56008	newly 5 weeks cumul	44803.	22819.4	{2000., 97600.}	95.0209	0.942775	newly 56 weeks cumul	56292.	25402.9	{2243., 103997.}	90.3169	0.964183
11	newly 3 weeks spec	8565.	661.26	{1., 1253.}	92.7979	2.28181	newly 3 weeks cumul	34772.	10791.4	{957., 42160.}	87.6646	0.948517	newly 5 weeks spec	4255.	360.27	{0., 820.}	94.0675	3.2794	newly 5 weeks cumul	45069.	11607.7	{957., 47985.}	84.4305	0.995849	newly 55 weeks cumul	54734.	13156.7	{957., 52696.}	82.0965	1.01441
12	newly 3 weeks spec	7136.	3141.18	{342., 11777.}	85.7405	0.817346	newly 3 weeks cumul	31568.	14100.1	{2586., 55604.}	86.8374	0.755772	newly 5 weeks spec	3139.	1649.02	{35., 4172.}	83.4317	0.873044	newly 5 weeks cumul	39318.	17984.8	{3260., 83164.}	85.503	0.7411	newly 56 weeks cumul	44760.	23858.7	{3848., 93356.}	82.992	0.671944
13	newly 3 weeks spec	7637.	3461.35	{455., 11841.}	83.7155	0.754516	newly 3 weeks cumul	33066.	17407.8	{2490., 56788.}	89.4943	0.721802	newly 5 weeks spec	3594.	1568.4	{148., 1843.}	90.2866	0.860064	newly 5 weeks cumul	41882.	21241.	{3126., 80286.}	86.148	0.721537	newly 57 weeks cumul	49021.	25294.9	{3457., 95200.}	84.593	0.710235
14	newly 3 weeks spec	8364.	3468.2	{406., 13399.}	81.7422	0.87419	newly 3 weeks cumul	30226.	12444.	{1676., 42972.}	80.6298	0.806998	newly 5 weeks spec	4850.	1627.28	{94., 5402.}	72.5249	0.908759	newly 5 weeks cumul	41750.	16408.2	{2418., 58080.}	77.5468	0.805912	newly 50 weeks cumul	54343.	23502.5	{3578., 76766.}	72.0684	0.74908
15	newly 3 weeks spec	7352.	1301.72	{340., 1557.}	85.46	0.969116	newly 3 weeks cumul	31019.	7150.2	{2483., 7810.}	84.3981	0.853337	newly 5 weeks spec	3380.	503.25	{47., 968.}	88.2186	1.12706	newly 5 weeks cumul	39368.	8404.07	{2932., 15141.}	83.424	0.86796	newly 51 weeks cumul	45493.	10141.6	{3247., 16943.}	82.4743	0.865828
16	newly 3 weeks spec	8547.	1342.01	{0., 4249.}	89.9933	2.06649	newly 3 weeks cumul	32656.	13545.4	{3091., 33729.}	82.5438	0.728622	newly 5 weeks spec	4015.	345.98	{0., 477.}	94.4005	2.3562	newly 5 weeks cumul	42788.	14548.1	{3270., 37043.}	82.1241	0.798115	newly 54 weeks cumul	49793.	15025.5	{3294., 37297.}	82.4168	0.843728
17	newly 3 weeks spec	6343.	1283.21	{27., 1330.}	101.3	1.66478	newly 3 weeks cumul	39322.	6446.09	{182., 9105.}	91.6275	1.3967	newly 5 weeks spec	1376.	371.59	{2., 132.}	111.674	1.97208	newly 5 weeks cumul	43673.	7556.16	{195., 10888.}	93.2267	1.42259	newly 60 weeks cumul	44678.	7898.51	{202., 11883.}	93.7239	1.42654
18	newly 3 weeks spec	6778.	806.75	{9., 508.}	98.5702	1.90428	newly 3 weeks cumul	35236.	4013.22	{206., 2914.}	94.7576	1.44794	newly 5 weeks spec	2583.	302.44	{0., 166.}	99.6647	2.54473	newly 5 weeks cumul	42014.	4821.47	{218., 3365.}	95.536	1.49342	newly 59 weeks cumul	45747.	5251.08	{221., 3552.}	95.8831	1.51827
19	newly 3 weeks spec	8875.	651.5	{0., 2633.}	92.6592	2.60919	newly 3 weeks cumul	35227.	6974.92	{494., 11584.}	85.9222	1.05535	newly 5 weeks spec	4524.	179.84	{0., 926.}	96.0248	2.88578	newly 5 weeks cumul	46101.	7492.86	{563., 14270.}	86.7687	1.13917	newly 54 weeks cumul	56361.	7712.61	{596., 15871.}	87.9273	1.21324
20	newly 3 weeks spec	6233.	1347.92	{17., 511.}	102.898	1.77453	newly 3 weeks cumul	33857.	5185.3	{262., 3419.}	94.5114	1.41478	newly 5 weeks spec	2013.	667.75	{0., 174.}	114.774	2.26812	newly 5 weeks cumul	39540.	6840.97	{293., 3845.}	95.9782	1.44752	newly 59 weeks cumul	42220.	7914.02	{300., 3953.}	97.5663	1.46389
21	newly 3 weeks spec	6457.	592.38	{19., 357.}	93.9418	1.70538	newly 3 weeks cumul	36224.	4539.45	{184., 3180.}	94.5973	1.4137	newly 5 weeks spec	1848.	154.53	{1., 86.}	98.4259	2.10566	newly 5 weeks cumul	41535.	4988.46	{184., 3534.}	93.8785	1.44385	newly 60 weeks cumul	43675.	5167.08	{191., 3685.}	93.5174	1.45721
22	newly 3 weeks spec	7395.	2591.76	{248., 8743.}	81.65	0.88106	newly 3 weeks cumul	32127.	19656.1	{3473., 57742.}	75.734	0.58773	newly 5 weeks spec	2882.	657.11	{7., 1323.}	87.8435	1.3133	newly 5 weeks cumul	39687.	21526.5	{3864., 58051.}	75.309	0.621207	newly 56 weeks cumul	44378.	23041.	{4032., 90757.}	75.2777	0.635764
23	newly 3 weeks spec	4881.	3867.7	{677., 13875.}	94.175	0.582181	newly 3 weeks cumul	26601.	15866.8	{3216., 43829.}	85.4831	0.622535	newly 5 weeks spec	1370.	2103.46	{177., 10608.}	135.165	0.466233	newly 5 weeks cumul	30613.	20825.6	{4236., 71970.}	88.7733	0.600039	newly 57 weeks cumul	31913.	25748.	{5200., 100138.}	91.0296	0.546187
24	newly 3 weeks spec	7357.	711.65	{16., 372.}	98.2456	1.84473	newly 3 weeks cumul	40276.	3700.41	{655., 1892.}	95.0333	1.47804	newly 5 weeks spec	2182.	247.17	{0., 149.}	99.4624	2.19933	newly 5 weeks cumul	46540.	4413.47	{655., 2245.}	94.8769	1.50073	newly 59 weeks cumul	48960.	4677.28	{655., 2563.}	94.9319	1.50622
25	newly 3 weeks spec	9005.	1005.83	{3., 1661.}	96.2793	2.19428	newly 3 weeks cumul	36976.	12509.6	{1253., 47866.}	89.0935	0.95839	newly 5 weeks spec	3489.	562.9	{0., 591.}	100.781	3.21585	newly 5 weeks cumul	46402.	13796.3	{1256., 48332.}	86.9286	1.0067	newly 55 weeks cumul	51783.	15730.3	{1263., 48478.}	87.0307	1.01566
26	newly 3 weeks spec	6466.	898.85	{109., 1599.}	95.5099	1.30483	newly 3 weeks cumul	27532.	8145.64	{1083., 6080.}	94.9188	1.01832	newly 5 weeks spec	2648.	268.95	{20., 198.}	98.9573	1.6756	newly 5 weeks cumul	34415.	8893.13	{1174., 8619.}	94.5507	1.06853	newly 53 weeks cumul	38357.	9278.91	{1219., 12748.}	94.2835	1.09442
27	newly 3 weeks spec	7481.	411.53	{107., 1089.}	94.499	1.46941	newly 3 weeks cumul	29075.	8188.75	{964., 8538.}	93.1691	0.99998	newly 5 weeks spec	4125.	111.24	{3., 296.}	97.3033	2.19957	newly 5 weeks cumul	38835.	8490.7	{1074., 8570.}	91.1495	1.07424	newly 53 weeks cumul	49190.	8763.21	{1147., 10852.}	89.9331	1.14199
28	newly 3 weeks spec	5303.	572.21	{34., 267.}	97.1116	1.5932	newly 3 weeks cumul	34205.	3750.65	{603., 3369.}	94.5231	1.30348	newly 5 weeks spec	965.	226.11	{1., 45.}	111.417	1.92186	newly 5 weeks cumul	37460.	4311.23	{643., 3703.}	94.168	1.31833	newly 60 weeks cumul	38276.	4835.81	{651., 3872.}	94.6021	1.32386
29	newly 3 weeks spec	9186.	2297.33	{407., 10231.}	83.7776	0.939343	newly 3 weeks cumul	36850.	12130.4	{2091., 24410.}	90.6863	0.900843	newly 5 weeks spec	4278.	1034.43	{129., 1238.}	90.2532	1.00775	newly 5 weeks cumul	47405.	14630.8	{2572., 46945.}	87.5417	0.899327	newly 57 weeks cumul	55913.	17605.9	{2968., 77126.}	86.0858	0.876938
30	newly 3 weeks spec	9408.	498.51	{0., 268.}	94.7012	2.22625	newly 3 weeks cumul	43928.	4220.64	{342., 7558.}	93.2381	1.44242	newly 5 weeks spec	3519.	205.86	{0., 88.}	98.3302	2.6881	newly 5 weeks cumul	53150.	4710.83	{383., 7558.}	92.8635	1.49765	newly 60 weeks cumul	58223.	5231.43	{397., 8350.}	92.6265	1.5272
31	newly 3 weeks spec	6334.	1332.11	{5., 2394.}	87.5991	1.31886	newly 3 weeks cumul	24601.	4753.93	{178., 6775.}	85.8503	1.09096	newly 5 weeks spec	3288.	1100.58	{0., 3512.}	84.2178	1.501	newly 5 weeks cumul	32650.	7035.09	{190., 12067.}	84.843	1.09081	newly 50 weeks cumul	39652.	17322.8	{190., 74008.}	87.5226	1.05427
32	newly 3 weeks spec	8242.	2729.41	{230., 12263.}	87.1921	1.0079	newly 3 weeks cumul	32101.	21561.1	{2314., 81103.}	93.909	0.663165	newly 5 weeks spec	4308.	604.55	{9., 904.}	93.0323	1.56525	newly 5 weeks cumul	42469.	23369.6	{2813., 94371.}	89.8438	0.722936	newly 56 weeks cumul	52787.	24173.5	{3224., 99752.}	86.3747	0.773898
33	newly 3 weeks spec	5633.	1740.09	{92., 2218.}	94.7213	1.08798	newly 3 weeks cumul	30340.	18500.9	{3435., 54388.}	90.9936	0.684667	newly 5 weeks spec	2012.	639.26	{2., 1024.}	100.836	1.73403	newly 5 weeks cumul	35798.	20141.2	{3447., 71700.}	89.597	0.707763	newly 60 weeks cumul	38479.	21367.9	{3447., 95904.}	89.0705	0.710388
34	newly 3 weeks spec	6383.	3579.64	{453., 13082.}	80.0003	0.650091	newly 3 weeks cumul	29792.	17009.1	{3490., 69678.}	85.5628	0.627747	newly 5 weeks spec	2446.	1825.4	{93., 4350.}	78.2788	0.635643	newly 5 weeks cumul	36225.	21290.	{4030., 86686.}	82.8568	0.601429	newly 56 weeks cumul	39694.	28089.5	{4661., 97855.}	79.5445	0.52062
35	newly 3 weeks spec	5611.	633.64	{41., 367.}	98.5721	1.7196	newly 3 weeks cumul	36155.	3752.69	{393., 4338.}	94.7521	1.39984	newly 5 weeks spec	1151.	192.44	{1., 55.}	102.549	2.02747	newly 5 weeks cumul	39920.	4288.44	{434., 4536.}	95.1241	1.42025	newly 60 weeks cumul	40865.	4575.98	{443., 4559.}	94.9878	1.4226
36	newly 3 weeks spec	9648.	2542.68	{262., 6710.}	81.3346	0.913679	newly 3 weeks cumul	37219.	16228.8	{2412., 26577.}	82.8767	0.712586	newly 5 weeks spec	5029.	932.64	{18., 1172.}	87.5244	1.27686	newly 5 weeks cumul	49093.	18639.9	{2704., 45437.}	81.2719	0.748645	newly 56 weeks cumul	60543.	20640.6	{3373., 66490.}	80.558	0.77302
37	newly 3 weeks spec	8031.	2524.67	{747., 4081.}	71.4303	0.669431	newly 3 weeks cumul	39903.	15362.8	{3380., 21217.}	84.0586	0.716973	newly 5 weeks spec	2840.	1187.02	{198., 2035.}	67.9859	0.651143	newly 5 weeks cumul	47625.	18158.	{4264., 25050.}	79.6327	0.67249	newly 57 weeks cumul	51821.	23200.6	{4869., 58069.}	74.3907	0.59489
38	newly 3 weeks spec	6443.	1537.45	{0., 3167.}	88.4912	1.55179	newly 3 weeks cumul	29746.	9573.63	{2452., 24413.}	77.8907	0.799131	newly 5 weeks spec	2643.	524.93	{0., 2217.}	89.0681	1.74162	newly 5 weeks cumul	36633.	11034.8	{2574., 28994.}	78.8889	0.840006	newly 54 weeks cumul	40381.	11838.7	{2588., 31349.}	78.8134	0.858391
39	newly 3 weeks spec	8056.	2392.46	{296., 7191.}	83.567	0.931473	newly 3 weeks cumul	34505.	17507.5	{2185., 24371.}	84.4736	0.693748	newly 5 weeks spec	3317.	859.13	{23., 1765.}	92.4941	1.28851	newly 5 weeks cumul	43007.	19708.1	{2613., 38769.}	84.0813	0.72824	newly 56 weeks cumul	48356.	22083.4	{2787., 90164.}	83.9676	0.736314
40	newly 3 weeks spec	7422.	3778.33	{534., 12981.}	90.8263	0.80166	newly 3 weeks cumul	30770.	17931.1	{2424., 72364.}	96.2094	0.772925	newly 5 weeks spec	3674.	1471.77	{155., 3126.}	90.6965	0.865206	newly 5 weeks cumul	39721.	21707.4	{2968., 88582.}	92.9058	0.775751	newly 57 weeks cumul	48338.	25175.2	{3812., 92618.}	88.6004	0.762109
41	newly 3 weeks spec	7711.	815.77	{54., 436.}	99.2529	1.68001	newly 3 weeks cumul	45986.	5543.95	{1182., 3189.}	96.3364	1.35504	newly 5 weeks spec	1635.	165.21	{1., 108.}	99.0783	2.0158	newly 5 weeks cumul	51212.	6072.29	{1202., 3500.}	96.6281	1.37902	newly 60 weeks cumul	52636.	6217.1	{1203., 3627.}	96.6537	1.38468
42	newly 3 weeks spec	7808.	1457.08	{129., 1545.}	99.3373	1.39512	newly 3 weeks cumul	27957.	4659.74	{961., 4617.}	92.5044	1.18083	newly 5 weeks spec	4650.	523.57	{17., 1018.}	91.4071	1.62827	newly 5 weeks cumul	38862.	6084.6	{1129., 7239.}	92.713	1.2336	newly 51 weeks cumul	51353.	7927.16	{1184., 18091.}	89.6036	1.25669
43	newly 3 weeks spec	8961.	3009.09	{198., 12582.}	83.5995	1.02536	newly 3 weeks cumul	34781.	22143.2	{1062., 82190.}	99.125	0.852803	newly 5 weeks spec	4598.	754.11	{34., 2164.}	86.0646	1.36123	newly 5 weeks cumul	46123.	24360.8	{1323., 96013.}	92.632	0.887553	newly 56 weeks cumul	56656.	25388.5	{1938., 102751.}	87.7489	0.906339
44	newly 3 weeks spec	7188.	2750.56	{289., 10670.}	89.0968	0.907397	newly 3 weeks cumul	31836.	13723.7	{2269., 51400.}	92.0025	0.808887	newly 5 weeks spec	3246.	1213.51	{30., 1852.}	87.6109	1.05315	newly 5 weeks cumul	39959.	16728.2	{2540., 77270.}	90.2989	0.811249	newly 56 weeks cumul	46072.	20256.5	{3086., 85547.}	86.5663	0.766762
45	newly 3 weeks spec	7528.	2960.91	{437., 3311.}	88.7175	0.783774	newly 3 weeks cumul	33385.	17150.3	{3834., 29917.}	87.8016	0.640646	newly 5 weeks spec	3187.	957.85	{37., 1778.}	83.4506	0.993884	newly 5 weeks cumul	41374.	19786.2	{4367., 31495.}	86.3842	0.658257	newly 58 weeks cumul	46827.	21344.8	{4620., 31555.}	84.3055	0.661635
46	newly 3 weeks spec	8312.	953.25	{23., 860.}	93.5802	1.72343	newly 3 weeks cumul	39284.	6112.83	{546., 9801.}	90.8788	1.33492	newly 5 weeks spec	3124.	224.	{2., 295.}	96.4693	2.21563	newly 5 weeks cumul	47611.	6795.1	{572., 10202.}	90.9967	1.38919	newly 60 weeks cumul	52255.	7016.85	{584., 10244.}	90.9573	1.4205
47	newly 3 weeks spec	8223.	718.5	{0., 894.}	98.4069	2.60413	newly 3 weeks cumul	34349.	16210.	{389., 67782.}	97.7541	1.16597	newly 5 weeks spec	3657.	314.55	{0., 428.}	99.3347	3.53958	newly 5 weeks cumul	43642.	16983.3	{428., 70012.}	92.5546	1.21385	newly 55 weeks cumul	49521.	17753.1	{438., 70012.}	90.3666	1.22459
48	newly 3 weeks spec	8405.	2926.39	{549., 4018.}	81.0535	0.74363	newly 3 weeks cumul	39417.	17349.	{5676., 22427.}	77.913	0.581048	newly 5 weeks spec	2763.	929.21	{45., 1961.}	74.6475	0.894081	newly 5 weeks cumul	47174.	19864.9	{6732., 24839.}	76.6074	0.58584	newly 58 weeks cumul	50734.	21527.	{7219., 26299.}	75.2744	0.575329
49	newly 3 weeks spec	6054.	3032.08	{381., 5486.}	82.0182	0.699614	newly 3 weeks cumul	27886.	14561.5	{3014., 63655.}	89.8522	0.706321	newly 5 weeks spec	2454.	1525.69	{54., 2854.}	73.1112	0.68746	newly 5 weeks cumul	34341.	18182.8	{3574., 64885.}	86.1733	0.675485	newly 56 weeks cumul	38035.	23602.2	{3893., 64950.}	78.9477	0.580282
50	newly 3 weeks spec	8264.	2805.36	{793., 4140.}	80.4393	0.738677	newly 3 weeks cumul	33965.	10388.9	{3379., 20355.}	77.0547	0.71438	newly 5 weeks spec	3752.	1643.56	{307., 2284.}	85.3097	0.733277	newly 5 weeks cumul	43375.	14213.1	{4370., 23281.}	77.2717	0.705662	newly 57 weeks cumul	50179.	18776.8	{5608., 29109.}	76.5827	0.659926
